# Comprehensive healthcare resource use among newly diagnosed congestive heart failure

**DOI:** 10.1186/s13584-017-0149-0

**Published:** 2017-06-05

**Authors:** Lori D. Bash, Dahlia Weitzman, Robert O. Blaustein, Ofer Sharon, Varda Shalev, Gabriel Chodick

**Affiliations:** 1 Merck & Co., Inc., Kenilworth, NJ USA; 2 Maccabitech, Maccabi Healthcare Services, Tel-Aviv, Israel; 3 Sackler Faculty of Medicine, Tel-Aviv University, Tel-Aviv, Israel; 4 MSD Israel, Hod Hasharon, Israel

**Keywords:** Congestive heart failure, Healthcare utilization, Costs of care, Community health

## Abstract

**Background:**

Congestive heart failure (CHF) is among the most common causes of hospital admissions and readmissions in the Western world. However, the burden of ambulatory care has not been as well investigated. The objective of this study was to assess the relative burden and direct medical costs of CHF including inpatient and outpatient care.

**Methods:**

We used longitudinal clinical data from a two-million member health organization in Israel (Maccabi Healthcare Services) to identify adults with newly diagnosed CHF between January 2006 and December 2012, either in the in- or outpatient setting. Adults without CHF were age- and sex-matched to CHF patients and healthcare utilization and all modes of healthcare costs were compared among them, excluding those in their last year of life.

**Results:**

The burden posed by 6592 CHF patients was significantly (*p* < 0.001) larger than that of 32,960 matched controls. CHF patients had significantly higher rates of baseline comorbidity and healthcare utilization compared to non-CHF controls. This was evident in all categories of healthcare services and expenses, including in- and outpatient visits, laboratory expenses, medication costs, among younger and older, men and women. Among those who incurred any healthcare costs, younger (45-64y) and older (65 + y) subjects with CHF were observed to have about 3.25 (95% CI: 2.96–3.56) and 2.08 (95% CI: 1.99–2.17) times the healthcare costs, respectively, compared to subjects without CHF after adjusting for patient characteristics.

**Conclusion:**

CHF is associated with an overall two- to three-fold higher cost of healthcare services depending on patient age, accounting for over half of all healthcare costs incurred by elderly CHF patients, and more than two-thirds of all costs among younger CHF patients. Observations of the large burden posed on one of the youngest societies in the developed world are profound, implicative of great opportunities to control the costs of CHF. Further research to understand how resource use impacts health outcomes and quality of care is warranted.

## Background

Congestive heart failure (CHF) is a major source of morbidity and mortality and is associated with both substantial health and economic costs. The prevalence of CHF among the adult population in the developed world is approximately 1–2%, rising to more than 10% among persons 70 years of age or older [1]. In Europe (European Society of Cardiology countries) alone, there are approximately 15 million patients with CHF [2], and in the US, more than 5.8 million [3, 4].

Prognosis among heart failure patients is not promising, with 1 year mortality rates of 28% observed in a local heart failure population [5]. While survival following heart failure diagnosis has been improving, among US Medicare patients, 1 year HF mortality was still at 29.6.% in recent years [6]. Thereafter, the mortality is near 10% per year according to English registry data. CHF patients continue to be at significant mortality risk, with a 5-year survival of 58%, compared to 93% in the age- and sex- matched general population [7]; even more recently, still about half of those diagnosed are not expected to survive more than 5 years in the US [8, 9].

Despite medical advances in the treatment of chronic CHF over the last 2 decades, heart failure accounts for a disproportionate number of hospitalizations. However, considerable international variability has been reported on the impact of CHF, and its case-fatality [10–12]. In the EuroHeart Failure survey, the median length of stay was 7–8 days, and 25% of patients were readmitted within 3 months [13]. In Canada, 13.9% of cardiovascular disease (CVD)-related hospitalizations, and 17% of days in hospital were due to heart failure [14]. Most (80–90%) CHF hospitalizations are due to worsening chronic HF, and few patients hospitalized with CHF present with de novo or end-stage CHF [15, 16].

Rehospitalizations among heart failure patients are common and portend worse prognosis [13, 14, 17–21]. In the US hospital-based OPTIMIZE-HF registry, patients reached a mortality rate of 8.6% within 60–90 days after hospital discharge, and nearly 30% of patients were rehospitalized overall [19]. In the Italian IN-HF Outcome registry, 1-year CHF re-hospitalization rates were slightly better at 20%, and CV mortality following hospitalization for worsening CHF reached 15–21% [21]. The multinational CHARM trials showed similar rates of hospitalization (19%), with about 3 times the hazard of death following CHF hospitalization [17].

CHF accounts for approximately 1–2% of the total healthcare expenditure in a number of industrialized countries [1]. In the US alone, CHF is associated with an estimated $29 billion in hospital charges annually [4], and $33.2 billion annually including direct and indirect costs [22]. In Israel, one of the youngest societies in the developed world, the relative cost is expected to be far greater than its Western counterparts (because of its young population, Israel would be expected to incur relatively minimal healthcare costs if not impacted by a burdensome disease such as CHF).

CHF is among the most common causes of hospital admissions and readmissions in Europe and the US, but there is a lack of such data from other regions. Variability in healthcare use and CHF outcomes by gender, has also been observed [11, 23, 24]. The aims of the present population-based study were to characterize and compare healthcare services utilization and costs between CHF patients with age- and sex-matched adults without CHF, with specific attention to gender and age differences.

## Methods

### Settings

The present retrospective cohort study was conducted using the computerized data of Maccabi Healthcare Services (MHS), a non-for-profit health organization providing full medical care to over 2-million members in Israel (~25% of the national population), and the second largest healthcare provider in the country. According to the Israeli National Health Insurance Act, MHS is obligated to provide care nationwide and to every citizen who wishes to join it. MHS’ central databases are automatically updated and include information on every service provided to members, including physician visits, dispensed medication, laboratory tests, nursing care, imaging, and hospital admissions. In addition, MHS maintains several automated patient registries, such as the diabetes mellitus [25], and cardiovascular diseases registries [26]. These registries are updated daily and automatically utilizing strict algorithms.

### Patient selection

Using MHS’ registry of cardiovascular patients, we selected all patients aged 21 or above that were diagnosed with CHF between January 2006 and December 2012 (study period), according to the following *International Classification of Diseases*, Ninth revision (ICD-9) codes of congestive heart failure (402.01, 402.11, 428.x, 514, 514.9, 518.4, 785.51). The earliest date of CHF diagnosis during the study period was defined as the study index date. To increase the cohort specificity, we included only patients with a CHF diagnosis made during at least two different hospitalizations or visits to cardiologists. A single diagnosis was sufficient to qualify a study patient if it was validated by a primary care physician, or if the patient had died within 2 months from the date of diagnosis.

Since the last year of life is considered to bear a disproportionately high economic toll on healthcare systems [27], we have focused on those who survived at least 1 year after diagnosis. This allowed for a more conservative comparison with non-CHF MHS members.

**Fig. 1:**
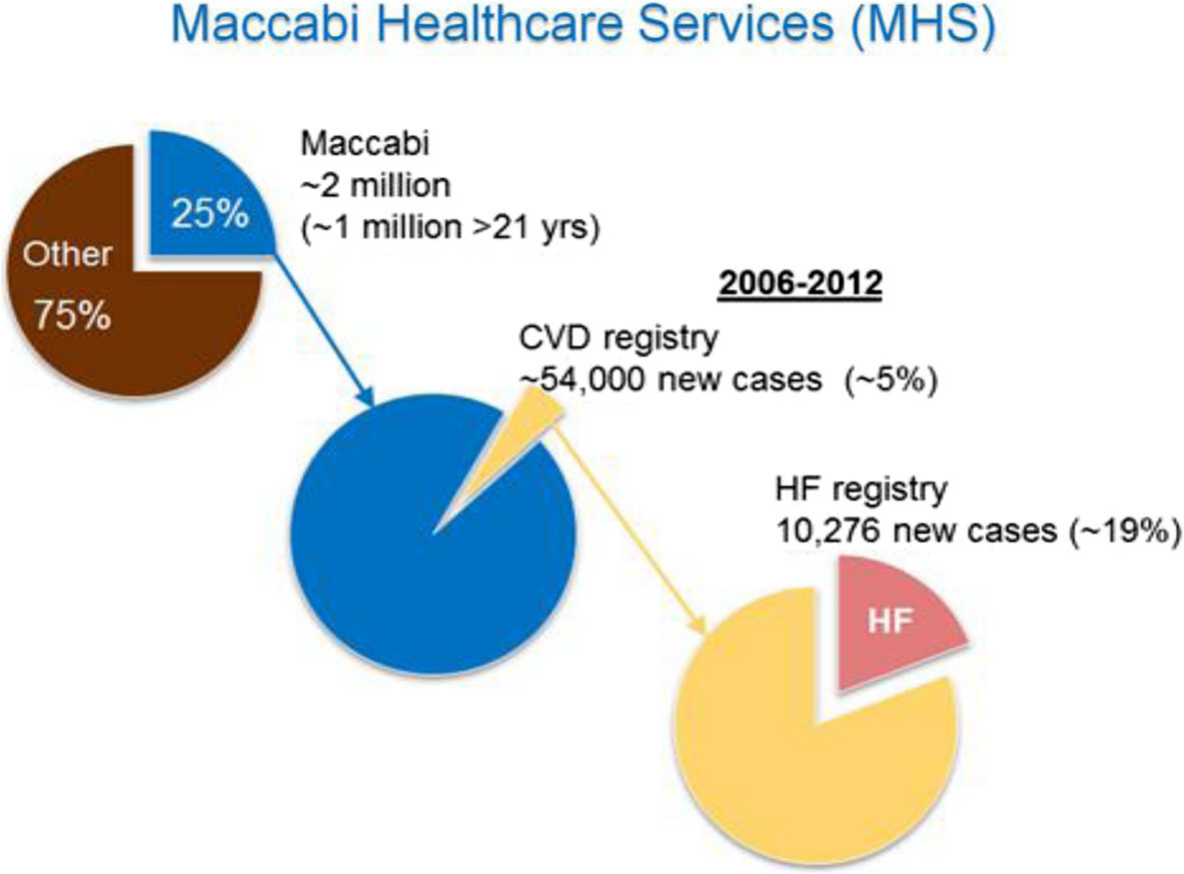
Study Sample. Attrition of CHF patients from the study sample: All adult MHS members (age ≥21 year) who entered MHS’ CHF registry between January 2006 and December 2012 were eligible for the current study. We assessed 6,592 patients among the 10,276 patients entering the CHF registry. A total of 3,684 were excluded due to ﻿one or more of the following: f﻿irst CHF indication not between 2006 and 2012; EF <40% reported >6 months before diagnosis; <1 year MHS enrollment before diagnosis; they had right heart failure; they did not have a prescription or dispensation of a diuretic; or exited MHS or died within the first year following diagnosis

We assessed 6,592 patients among the 10,276 entering the CHF registry; a total of 3,684 were excluded (Fig. 1). To ensure only incident CHF cases were captured, we excluded patients (*n* = 490) whose first CHF indication was not between 2006 and 2012, patients with history (more than 6 months prior to index date) of ejection fraction (EF) of less than 40% (*n* = 338), patients who were MHS members for less than 1 year prior to the index date (*n* = 543) or left MHS within 1 year from index date (*n* = 23). To increase specificity of the cohort, we excluded those that were never prescribed with diuretics (*n* = 690), an essential component of current treatment in heart failure [28]. We also excluded patients with right heart failure (*n* = 18), as well as 1,582 patients that died within 1 year of CHF diagnosis.

The 6,592 CHF patients surviving at least 1 year post-diagnosis, were compared to 32,960 age and sex-matched MHS members without a diagnosis of CHF. Matching was done in a 1 to 5 ratio with ±1 year of age among subjects without CHF who also had at least 1 year of MHS enrollment and were members for at least 1 year after index.

### Data collection

All demographic, administrative and clinical data were collected from MHS’ computerized databases. These included, but were not limited to demographic and administrative data (age, sex, MHS enrollment date, date of death (follow-up for all-cause mortality continued to 1st June 2014), smoking status), comorbidity based on MHS chronic disease registries, patient’s socioeconomic status (SES) and healthcare utilization data.

The following comorbidities at index date were identified through MHS registries: diabetes mellitus [25, 29], hypertension [30], chronic kidney disease (CKD), chronic obstructive pulmonary disease (COPD), and specific CVD registry [26] diagnoses: cerebrovascular accident (CVA), transient ischemic attack (TIA), myocardial infarction (MI), peripheral vascular disease (PVD) and atrial fibrillation (AF). SES was defined according to the poverty index of the member’s enumeration area as defined during the Israel national census in 2008 (or 1995 for those deceased prior to 2008). The poverty index was based on several parameters, including household income, education, crowding, material conditions, and car ownership, and ranges from 1 to 20, based on cluster analysis, with 1 being the lowest and 20 being the highest SES level [31]. Healthcare services utilization data including, but not limited to, dates of visits to primary and secondary physicians, specialty of the treating physician, and hospitalization dates were extracted. The cost of healthcare services is determined in several ways depending on type of service. Some services incur costs which are determined internally in MHS (e.g., cost of secondary care consultation) while others are determined by the ministry of health (e.g., hospitalizations) or via contracts with external suppliers (e.g., payment per medications) [32]. In addition, MHS defines members’ copayment for each service, with decreased copayments for specific welfare-recipient populations. MHS’ actual expenditure per member was extracted from an automated database used for administrative purposes, where all financial transactions referring to each MHS member are summed by category of services on a monthly basis. The expenditure categories are defined by MHS’ administrative considerations, and include the following: 1) MHS primary and secondary physicians, 2) secondary specialized clinics, which are either private clinics providing secondary care, or, either private or MHS institutes, providing services other than physician consultation (e.g., diagnostic imaging, ambulatory procedures, etc.), 3) laboratory tests, 4) dispensed medications (excluding over-the-counter drugs), 5) all hospital-related expenses, including outpatient, ER and in-patient expenses, 6) other expenses (e.g., ambulance transportation, medical devices and accessories, medical nutrition, etc.), and 7) total monthly expenses per patient.

### Statistical analyses

The index date for CHF diagnosis was defined as the first diagnosis by a primary care physician, cardiologist, or hospital discharge letter. Non-CHF controls were assigned an average index date according to their age- and sex - matched group.

For continuous variables, mean and standard deviation (SD) or median and interquartile range (IQR) were calculated. Characteristics were compared between CHF patients and non-CHF controls using the Chi-square test or Mann-Whitney test for categorical or continuous variables, respectively.

The monthly cost of healthcare services per individual member (overall and by service category) was available from 2007 forward. Costs were analyzed for 5,407 CHF patients and 27,035 matched non-CHF controls aged 45 to 94 years old with index dates between 2007 and 2012.

We compared direct costs of healthcare services in the year following diagnosis between CHF patients and non-CHF controls in multiple ways. First, the ratio of the mean cost per patient per type of service, by subject group compared to the mean cost of physician visits of non-CHF subjects (reference group) was calculated. Patients in the top percentile of costs were excluded as outliers. Secondly, a 2-step approach was applied where a) we estimated the relative risk that an individual with, compared to an individual without CHF, bore any cost for MHS per category of healthcare services, and, b) among those who had incurred costs, the ratio of costs between those with and without CHF was estimated using generalized linear models, with gamma distribution and log-link function. These models adjusted for residual confounding by sex and age, and for confounding by SES, AF, MI, non-MI ischemic heart disease (IHD), CVA, TIA, PVD, diabetes mellitus, hypertension, and CKD (with and without dialysis). Patients within the upper one percentile of costs were excluded from both steps of this analysis as well. Analyses were done in IBM SPSS Statistics for Windows, Version 22.0 (Armonk, NY: IBM Corp).

## Results

A total of 6,592 CHF patients surviving at least 1 year from diagnosis were compared to 32,960 age- and sex-matched MHS members without a diagnosis of CHF who also had at least 1 year of enrollment in MHS and were members for at least 1 year after index. Nearly three quarters of CHF patients were over 65 years of age (Table 1), very different from the general population (Appendix 1). On average, women were older, about 76 years old, compared to men who were about 70 years old, and much less likely to smoke (Table 1). In both men and women, CHF patients were more likely to have a history of smoking, and have slightly lower SES. Among these patients, only 42% of women, and 51% of men performed an echo test near time of diagnosis, and among these 37%, 16% and 47% had HF with reduced ejection fraction (EF < 40%; HFrEF), intermediate EF (EF = 40–49%) and preserved (EF ≥ 50%; HFpEF), respectively (data not shown). Due to the large degree of missing information, we did not distinguish between heart failure type in cost analyses.

**Table 1:** Baseline^a^ characteristics of the study group by gender

	Overall		Females		Males	
	CHF patients	Non-CHF age & sex matched MHS members	CHF patients	Non-CHF age & sex matched MHS members	CHF patients	Non-CHF age & sex matched MHS members
N	6,592	32,960	2,816	14,080	3,776	18,880
Female	42.7%	42.7%				
Age, mean ± SD, y	72.6 ± 12.4*	72.6 ± 12.5*	75.9 ± 11.5	76.0 ± 11.5	70.1 ± 12.5*	70.1 ± 12.5*
Age ≥ 65 years, %	74.0%	74.0%	84.1%	84.1%	66.5%	66.5%
SES, mean ± SD	11.6 ± 4.2	12.4 ± 4.2	11.5 ± 4.2	12.3 ± 4.2	11.6 ± 4.3	12.5 ± 4.2
Ever smoked, % (n)	17.3% (1,138)	12.5% (4,112)	7.5% (211)	6.8% (953)	24.5% (927)	16.7% (3,159)
Hypertension	83.8%	64.5%	89.1%	71.9%	79.9%	59.0%
CKD	59.2%	30.2%	65.3%	35.1%	54.7%	26.7%
Diabetes mellitus	45.9%	23.8%	45.4%	23.3%	46.2%	24.1%
AF	37.0%	8.1%	45.3%	8.3%	34.0%	8.0%
Non-MI IHD	33.4%	12.5%	30.8%	8.9%	35.2%	15.3%
MI	36.9%	8.1%	23.4%	4.9%	47.0%	10.4%
Cancer	20.9%	18.5%	21.1%	17.1%	20.8%	19.5%
CVA	12.7%	5.2%	12.9%	5.2%	12.6%	5.1%
PVD	14.3%	4.2%	9.1%	2.8%	18.2%	5.3%
TIA	6.3%	2.4%	6.0%	2.3%	6.6%	2.4%
On dialysis	1.79%	0.34%	1.2%	0.2%	2.3%	0.5%
Health services utilization in year following diagnosis						
Primary physician^b^	19 (12, 29)	8 (4, 14)	19 (12, 29)	9 (5, 15)	19 (12, 29)	8 (4, 13)
Cardiology services^b^	11 (5, 22)	3 (1, 7)	8 (3, 18)	3 (1, 6)	13 (6, 25)	3 (1, 7)
All non-cardiology medical specialties^b^	29 (14, 57)	17 (7, 34)	29 (14, 57)	17 (7, 34)	29 (14, 57)	16 (7, 33)
Hospitalization days, Median (IQR)	5 (1, 16)	0 (0, 0)	6 (1, 17)	0 (0, 0)	5 (1, 15)	0 (0, 0)
% (n) with > =1 hospitalization	77.8% (5127)	20.5% (6766)	78.3% (2205)	21.2% (2981)	77.4% (2922)	20.0% (3785)
% (n) with > =1 ER visit	11.1% (730)	5.2% (1708)	11.8% (332)	5.4% (767)	10.5% (398)	5.0% (941)

*All *p* < 0.001 between CHF patients and non-CHF controls, except for overall and male age: *p* = 0.555 and *p* = 1.00, respectively, and cancer in males: *p* = 0.066

^a^comorbid conditions diagnosed before index or within one year from index among CHF and non-CHF subjects (*N* = 39,552)

^b^Median (IQR) visits in year following diagnosis

Compared to their non-CHF controls, CHF patients were more likely (*p* < 0.001) to have several types of comorbid conditions (including hypertension, CKD, diabetes, AF, IHD, history of MI, CVA, PVD, TIA). This was true among both men and women, with the exception of cancer, which was numerically, though not statistically significantly, more often experienced by male CHF patients (*p* = 0.066) (Table 1). Within the year following diagnosis, CHF patients pose a much greater weight of healthcare services compared to their counterparts without CHF. Discrepancies in the presence of comorbidities between those with and without CHF became increasingly evident in the year after diagnosis as patients experienced more healthcare interactions and diagnoses (data not shown).

We observed significantly higher rates of all types of healthcare services utilization and costs among CHF patients compared to their non-CHF controls among both men and women. Most (~78%) CHF patients were hospitalized at least once, compared to a minority (~21%) of non-CHF adults (Table 1). Female and male CHF patients spent 6 and 5 (median) days respectively, in the hospital and experienced 19 (median) primary physician visits during the year following diagnosis, compared to 0 hospitalization days, and 9 and 8 physician visits respectively, among their female and male non-CHF controls. ER visits, specialty care visits, and other secondary services were also substantially higher among both male and female CHF patients compared to their non-CHF counterparts.

Among older and younger age groups in both men and women, those with CHF, compared to those without CHF consistently experienced higher costs for all types of healthcare (Table 2). Hospital-related costs were by far the highest type of costs. Compared to the costs of physician visits among patients without CHF, hospital-related costs were 4–7 times more among those without, and 23–47 times more among those with, CHF. Differences in laboratory costs were less pronounced between those with and without CHF, but 2–3 fold differences were still observed. Overall costs were substantially higher among those with, compared to those without CHF, for both age groups, though more pronounced differences were observed among younger males.

**Table 2:** Ratio of mean cost of various healthcare services of CHF and non-CHF patients vs. the mean cost of non-CHF primary and secondary MHS physician visits by age group

Type of costs	45–64 years				65–94 years			
	Ratio of mean costs of healthcare services of CHF and non-CHF vs. the mean cost of non-CHF physician visits^a^				Ratio of mean costs of healthcare services of CHF and non-CHF vs. the mean cost of non-CHF physician visits^a^			
	Females *N* = 2014		Males *N* = 5946		Females *N* = 11667		Males *N* = 12492	
	CHF	non-CHF	CHF	non-CHF	CHF	non-CHF	CHF	non-CHF
Primary & secondary MHS physicians	2.2	1.00 (reference)	2.5	1.00 (reference)	1.6	1.00 (reference)	1.7	1.00 (reference)
Laboratory tests	0.4	0.2	0.3	0.1	0.3	0.2	0.3	0.1
Other	1.3	0.7	1.6	1.0	1.1	0.8	1.1	1.0
Secondary clinics	6.8	1.8	6.0	1.8	4.2	2.4	4.4	2.5
Medications	5.7	1.1	8.0	1.2	4.8	1.4	4.6	1.4
Hospital-related costs	37.3	4.0	46.7	5.1	23.3	6.0	26.2	7.1
Total expenses	56.3	8.9	74.4	10.0	34.4	11.2	41.3	12.7

^a^Patients within the upper 1 percentile of costs per category of services were excluded from the analysis; Ratio of costs of reference categories: Among non-CHF controls, the mean cost of physician visits of the 65–94 age group was 1.12 and 1.61 times that of the 45–64 age group among females and males, respectively. Within age groups, the mean cost of physician visits of female was 1.33 and 0.93 times that of males, in the 45–64 and 65–94 age groups, respectively

CHF patients were more likely to have incurred any type of healthcare cost in the year following diagnosis compared to their non-CHF counterparts. The most pronounced difference between those with and without CHF was incurring hospital-related costs both among patients aged 45–64 years old (OR = 1.78) and older patients (OR = 1.41) (Table 3).

**Table 3:** Relative risk for incurring costs among all patients and crude and adjusted ratio of costs (95% CI) *among patients whom had incurred costs* of CHF patients vs non-CHF controls, 2007–2012*

	45–64 years (*N* = 8,040) (CHF *N* = 1,340; non-CHF *N* = 6,700)									
	Relative risk for incurring healthcare costs among all patients (*N* = 8,040)				Females (*N* = 2,034)			Males (*N* = 6,006)		
Type of cost	RR^a^ of incurring costs (95% CI)	N incurring costs	Crude ratio (95% CI)	Adjusted ratio (95% CI)	N incurring costs	Crude ratio (95% CI)	Adjusted ratio (95% CI)	N incurring costs	Crude ratio (95% CI)	Adjusted ratio (95% CI)
Primary & secondary MHS physicians	1.10 (1.09–1.11)	7,236	2.08 (1.98–2.18)	1.66 (1.56–1.76)	1,909	2.11 (1.94–2.30)	1.80 (1.61–2.01)	5,400	2.23 (2.10–2.36)	1.72 (1.61–1.85)
Laboratory tests	1.27 (1.25–1.29)	6,292	1.76 (1.67–1.85)	1.43 (1.34–1.52)	1,691	1.84 (1.67–2.03)	1.42 (1.25–1.62)	4,568	1.57 (1.52–1.63)	1.33 (1.27–1.38)
Secondary clinics*	1.31 (1.29–1.33)	6,186	5.35 (4.96–5.76)	3.62 (3.32–3.94)	1,645	3.81 (3.32–4.37)	2.67 (2.27–3.13)	4,532	5.94 (5.42–6.50)	3.70 (3.34–4.09)
Medications	1.15 (1.14–1.17)	7,052	3.01 (2.80–3.24)	1.76 (1.62–1.90)	1,880	3.50 (3.04–4.04)	2.32 (1.95–2.76)	5,173	2.83 (2.60–3.08)	1.68 (1.53–1.85)
Hospital-related costs	1.78 (1.74–1.83)	4,872	5.05 (4.57–5.58)	2.86 (2.56–3.19)	1,481	6.35 (5.21–7.74)	3.52 (2.85–4.35)	3,392	4.71 (4.19–5.29)	2.61 (2.29–2.98)
Other medical expenses	1.38 (1.33–1.42)	3,110	1.54 (1.40–1.70)	1.40 (1.24–1.57)	892	1.69 (1.40–2.04)	1.51 (1.19–1.91)	2,236	1.50 (1.34–1.69)	1.36 (1.18–1.57)
Total	1.11 (1.10–1.11)	7,279	6.38 (5.87–6.93)	3.25 (2.96–3.56)	1,906	5.87 (5.02–6.86)	3.17 (2.68–3.75)	5,350	6.39 (5.8–7.05)	3.32 (2.98–3.70)
	65–94 years (*N* = 24,402) (CHF *N* = 4,067; non-CHF *N* = 20,335)									
	Relative risk for incurring healthcare costs among all patients *N* = 24,402				Females (*N* = 11,784)			Males (12,618)		
Type of cost	RR^a^ of incurring costs (95% CI)	N incurring costs	Crude ratio (95% CI)	Adjusted ratio (95% CI)	N incurring costs	Crude ratio (95% CI)	Adjusted ratio (95% CI)	N incurring costs	Crude ratio (95% CI)	Adjusted ratio (95% CI)
Primary & secondary MHS physicians	1.07 (1.07–1.08)	22,171	1.50 (1.46–1.54)	1.32 (1.29–1.36)	10,701	1.46 (1.41–1.52)	1.32 (1.26–1.37)	11,693	1.63 (1.57–1.68)	1.40 (1.35–1.45)
Laboratory tests	1.12 (1.12–1.13)	21,430	1.61 (1.56–1.65)	1.34 (1.30–1.38)	10,370	1.66 (1.57–1.77)	1.40 (1.30–1.51)	11,117	1.65 (1.59–1.72)	1.37 (1.32–1.43)
Secondary clinics*	1.14 (1.13–1.15)	21,144	3.07 (2.95–3.19)	2.26 (2.16–2.35)	10,102	3.06 (2.90–3.24)	2.23 (2.10–2.37)	10,988	3.07 (2.91–3.25)	2.10 (1.98–2.23)
Medications	1.07 (1.07–1.08)	22,771	1.64 (1.58–1.69)	1.32 (1.27–1.37)	11,047	1.63 (1.56–1.7)	1.34 (1.27–1.41)	11,725	1.62 (1.54–1.70)	1.26 (1.19–1.33)
Hospital-related costs	1.41 (1.40–1.43)	17,568	2.63 (2.49–2.78)	1.94 (1.83–2.05)	8,507	2.72 (2.51–2.94)	1.99 (1.83–2.17)	9,062	2.58 (2.39–2.78)	1.91 (1.76–2.07)
Other medical expenses	1.16 (1.14–1.18)	12,826	1.24 (1.17–1.30)	1.19 (1.12–1.26)	6,299	1.29 (1.19–1.39)	1.24 (1.14–1.36)	6,593	1.16 (1.07–1.25)	1.11 (1.02–1.21)
Total	1.07 (1.06–1.07)	22,810	2.99 (2.87–3.11)	2.08 (1.99–2.17)	11,039	2.89 (2.72–3.06)	1.95 (1.84–2.08)	11,719	2.93 (2.76–3.11)	2.06 (1.94–2.19)

*ALL *p*-values are <0.001

^a^RR: relative likelihood that a subject with CHF, compared to a subject without CHF would incur any type of healthcare cost

Note: 1) The upper 1 percentile of costs within each age and sex group and service type were excluded;

2) A total of 185 patients were on dialysis at index or initiated dialysis in the first year post index. These patients were excluded from analysis of secondary clinics costs (61 and 124 had incurred costs in the upper 1 percentile and lower 99%, respectively), and from total costs analysis (94 and 91 had incurred costs in the upper 1 percentile and lower 99%, respectively);

3) All models adjusted for sex, age, SES, AF, MI, non-MI IHD, CVA, TIA, PVD, Diabetes mellitus, hypertension, and CKD (with and without dialysis)

Similarly, among those who had incurred healthcare costs in the various service categories, the ratio of costs between those with and without CHF was generally higher among younger patients than older ones and similar between genders. After adjustment for clinical and demographic characteristics, younger patients still showed more than triple (3.25 (2.96–3.56)), and older patients, double (2.08 (1.99–2.17)), the total healthcare costs of their non-CHF counterparts, respectively. Prevalent cases of CHF made up approximately 1.2% of adult MHS enrollees in 2009 (midpoint of study period) and about 7.6% of the total direct expenditure; in 2015, cases made up only 0.85% of enrollees and 4.4% of expenditure.

Differences in cost incurred between study groups were also most pronounced in hospital and secondary clinic costs, more so among younger patients (Table 3). Significant gender differences were found only among younger patients (p for interaction <0.05). The relative costs (95% CI) between those with and without CHF was more discrepant among younger women compared to younger men, where adjusted in-hospital related costs were 3.52 (2.85–4.35) and 2.61 (2.29–2.98) times, respectively. The same was observed for medication costs, 2.32 (1.95–2.76) vs. 1.68 (1.53–1.85)), though the converse was true for secondary clinic costs: 3.70 (3.34–4.09) among males and 2.67 (2.27–3.13) among females.

## Discussion

The results of the present analysis show that CHF is associated with substantial healthcare utilization and costs incurred during the year following diagnosis, despite the relatively young population of Israel (MHS median age 42 (Appendix 1)) .

Consistent with previous reports [1, 4, 12], the healthcare resource utilization, including economic cost and weight on services exerted by CHF patients, was significantly larger than that of sex- and age-matched subjects without CHF. This was evident in primary, secondary, and tertiary services including cardiac and non-cardiac services, all types of expenses (medications, lab tests, etc.), among both men and women, and in both younger and older age groups. However, even after controlling for differences in comorbidities in both groups among only patients who had incurred any healthcare costs, the burden of CHF remains high, with total costs of CHF patients being 2–3 times higher than non-CHF controls. In fact, among older (65–94 years) CHF patients, 52% of all healthcare costs incurred are due to CHF alone, and among younger (45–64 years) patients, an even greater proportion, with CHF accounting for 69% of all costs.

While considerable resources are expended on CHF patients of both sexes, some variations between age groups were observed. When comparing the likelihood of CHF patients incurring *any* cost on specific types of healthcare services, to that of non-CHF patients, younger patients tended to have higher relative likelihood of incurring costs than older patients in most types of health services. Among those who incurred any cost on various healthcare services, the ratios of costs between CHF and non-CHF patients were higher among younger CHF patients than older patients, and among younger patients, some sex differences were observed. Compared to women, men had higher CHF to non-CHF ratios of secondary clinic costs, while females had higher ratios in hospital-related costs. This is reflected in the higher frequency of cardiology clinic visits for males vs. females with CHF and vice versa for days of hospitalization, while no difference was observed in the non-CHF group.

Overall observations with regard to relative costs among those with and without CHF were very similar to those found in other populations [12], demonstrating substantially higher costs incurred by those with CHF compared to those without CHF for all types of healthcare use. Not surprisingly, while costs were greater among the older population, the ratio of costs between those with and without CHF was more pronounced among the younger (45–64 years) adults, who have fewer comorbidities, and so have very few non-CHF related healthcare costs.

The ratio of healthcare costs borne by those with and without CHF was very similar regardless of sex and is larger among younger patients, not unexpected since older non-CHF enrollees have a higher baseline cost compared with younger enrollees. Relative costs provide a valuable perspective on costs incurred by CHF patients relative to their sex and age-matched counterparts; findings were consistent with other populations [12], and may be more generalizable to others with similar demographics.

Because of the young population of MHS, comparable to the nation’s make-up, CHF poses an even greater onus to Israel than that observed in other industrialized nations [1]. As one of the youngest populations in the developed world, CHF was observed to account for 2–4 times the proportion of all healthcare expenditures compared to the proportions seen in other developed nations (1–2% in Netherlands, New Zealand, Scotland, Spain, Sweden, and the US) [1]. Prevalent cases of CHF made up approximately 1.2% of adult MHS enrollees in 2009 (the midpoint of the study period), and 0.85% in 2015. In 2009, about 7.6% of the total direct expenditure on adult patients was spent on the population of CHF prevalent cases, on average, 6.7 times the average costs of a non-CHF patient. In 2015, due to an aging population (Appendix 1), these figures decreased to 4.4% of the total expenditure, and on average 5.4 times the average costs of a non-CHF patient. While Maccabi Healthcare Services (together with the Gertner Institute) has been involved in opening one of the first telemedicine centers of its kind to actively monitor HF patients [33], part of a unique intervention to reduce costs and improve quality of care in HF (and other chronic conditions), it has had modest results with regard to patient outcomes [34]. Early reports have shown promising results for future cost savings and a cost-effective approach [35]; in light of the observations here, the cost/benefits of this disease management intervention along with other efforts should continue to be evaluated as the program continues to mature.

The present study has a number of strengths, among them, the population-based cohort, systematic and comprehensive collection of individual-level data, including socio-demographic information, medical history, and laboratory data which decreases the possibility for bias from study outcomes, as well as in- and out-patient clinical and cost information. Compared to previous local observational studies such as the Heart Failure Survey in Israel (HFSIS) [5], we were able to capture updated comprehensive in- and outpatient data as well as medical history on nearly twice the patients with relative information on their counterparts without CHF. Inherently, observational studies are prone to some biases, including unmeasured or mis-measured covariates. Missing and undocumented echo examinations are very common in clinical practice [36], though this is a limitation of this data. While the present analyses did adjust for SES, this was based on the poverty index of patients’ enumeration area, not on individuals. These measures may not adequately control for individual resources or other indicators of access to healthcare resources, so residual confounding may be possible. In the current analyses, we focused only on a single year of costs which may underestimate the true healthcare costs and utilization experienced by patients with CHF, a chronic progressive disease. This approach is conservative and as such, does not reflect the burden of last year of life for most patients, which may show exaggerated costs compared to their non-CHF counterparts.

Despite limitations inherent to the nature of the database, these findings provide a perspective on the substantial health and economic costs borne by Israeli patients with CHF and the health systems supporting them, an even greater relative cost than observed in other developed populations [1, 2, 12, 13].

## Conclusions

Disease management in heart failure continues to challenge healthcare systems globally; how to reduce the substantial resource use without negatively impacting patient outcomes, and better understand the observed gender differences requires further investigation. Our observations underscore the considerable healthcare strain of CHF patients, apparent even more so in this Israeli population, one of the youngest societies in the developed world. As younger nations become increasingly developed and industrialized, they will experience an increase in chronic disease. As they do, healthcare systems will bear relatively large healthcare expenditures for relatively less common morbidities. We observed that here, as CHF poses a substantial burden on the healthcare system, and an even greater relative cost on the healthcare expenditures of a young population. It is critical to note that because the *relative* burden that CHF poses on young populations is far greater than that posed on aging populations, so too are the potential gains to be made by improving the early diagnosis and treatment of CHF.

While quality compensation, or pay-for-performance programs are on the rise, where heart failure specifically, (in the US and other areas), is among the targets of several quality initiatives, similarly incentivized programs do not currently exist within the Israeli health system which generally operates on a per diem basis. Evidence suggests that there is potential for health care improvement as a result of these programs [37] though findings have not been consistent [38, 39]. Observations seen here may have implications with regard to the potential for improvement of healthcare system efficiencies, as it relates to quality initiatives and incentives for improvement in patient care. Further research is warranted to understand the factors that are independently and significantly associated with healthcare utilization and whether and how increased resource use affects health outcomes and quality of care among these patients. Together with the existing telemedicine program [33, 35] that was expanded from its initial pilot [34] to become part of routine services offered to HF patients, additional efforts to understand how to optimize both patient outcomes and reduce costs are needed. While all populations will benefit from early detection and aggressive disease management that are essential to control the toll CHF takes on the patient, their caretakers, and the health systems supporting them, the opportunity for relative improvement of the systems supporting young populations is even greater.

## Figures and Tables

**Fig. 2:**
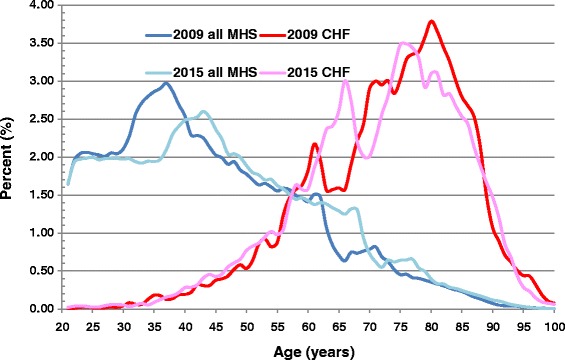
Population age distribution in Maccabi Health Services: All members and those with CHF, 2009 and 2015. MHS median (IQR) age among adults ≥21 years old: 2009: 42.2 (33.1–55.5) years; 2015: 44.8 (33.9–58.1) years

## Abbreviations

AF: Atrial fibrillation
CHF: Congestive heart failure
CKD: Chronic kidney disease
COPD: Chronic obstructive pulmonary disease
CVA: Cerebrovascular accident
CVD: Cardiovascular disease
EF: Ejection fraction
ER: Emergency room
ICD-9: International Classification of Diseases, Ninth revision
IHD: Ischemic heart disease
IQR: Interquartile range
MHS: Maccabi Healthcare Services
MI: Myocardial infarction
OR: Odds ratio
PVD: Peripheral vascular disease
SD: Standard deviation
SES: Socioeconomic status
TIA: Transient ischemic attack
US: United States

## References

[1] McMurray JJ, Adamopoulos S, Anker SD (2012). ESC guidelines for the diagnosis and treatment of acute and chronic heart failure 2012: The Task Force for the Diagnosis and Treatment of Acute and Chronic Heart Failure 2012 of the European Society of Cardiology. Developed in collaboration with the Heart Failure Association (HFA) of the ESC. Eur J Heart Fail.

[2] Ambrosy AP, Fonarow GC, Butler J, Chioncel O, Green SJ (2014). The Global Health and Economic Burden of Hospitalizations for Heart Failure. J Am Coll Cardiol.

[3] Lloyd-Jones D (2010). Heart disease and stroke statistics-2010 update: a report from the American Heart Association. Circulation.

[4] Roger VL, Go AS, Lloyd-Jones DM, Adams RJ, Berry JD, Brown TM, Carnethon MR, Dai S, de Simone G, Ford ES, Fox CS, Fullerton HJ, Gillespie C, Greenlund KJ, Hailpern SM, Heit JA, Ho PM, Howard VJ, Kissela BM, Kittner SJ, Lackland DT, Lichtman JH, Lisabeth LD, Makuc DM, Marcus GM, Marelli A, Matchar DB, McDermott MM, Meigs JB, Moy CS, Mozaffarian D, Mussolino ME, Nichol G, Paynter NP, Rosamond WD, Sorlie PD, Stafford RS, Turan TN, Turner MB, Wong ND, Wylie-Rosett J (2011). American Heart Association Statistics Committee and Stroke Statistics Subcommittee. Heart disease and stroke statistics-2011 update: a report from the American Heart Association. Circulation.

[5] Garty M, Shotan A, Gottlieb S, Mittelman M, Porath A, Lewis BS, Grossman E, Behar S, Leor J, Green MS, Zimlichman R, Caspi A (2007). HFSIS Steering Committee and Investigators. The management, early and one year outcome in hospitalized patients with heart failure: a national Heart Failure Survey in Israel-HFSIS 2003. Isr Med Assoc J.

[6] Chen J, Normand SL, Wang Y, Krumholz HM (2011). National and regional trends in heart failure hospitalization and mortality rates for Medicare beneficiaries, 1998–2008. JAMA.

[7] Hobbs FD, Roalfe AK, Davis RC (2007). Prognosis of all-cause heart failure and borderline left ventricular systolic dysfunction: 5 year mortality follow-up of the Echocardiographic Heart of England Screening Study (ECHOES). Eur Heart J.

[8] Roger VL, Weston SA, Redfield MM, Hellermann-Homan JP, Killian J, Yawn BP, Jacobsen SJ (2004). Trends in heart failure incidence and survival in a community-based population. JAMA.

[9] National Center for Health Statistics. Mortality multiple cause micro-data files: public-use data file and documentation: NHLBI tabulations. 2011. http://www.cdc.gov/nchs/products/nvsr.htm. Accessed 19 May 2015.

[10] West R, Liang L, Fonarow GC, Kocio R, Mills RM, O’Connor CM, Hernandex AF (2011). Characterization of heart failure patients with preserved ejection fraction: a comparison between ADHERE-US registry and ADHERE International registry. Eur J Heart Fail.

[11] Rodriguez F, Wang Y, Johnson CE, Foody JM (2013). National Patterns of Heart Failure Hospitalizations and Mortality by Sex and Age. J Card Fail.

[12] Corrao G, Ghirardi A, Ibrahim B, Merlino L, Maggioni AP (2014). Burden of new hospitalization for heart failure: a population-based investigation from Italy. Eur J Heart Fail.

[13] Cleland JG, Swedberg K, Follath F (2003). The EuroHeart Failure survey programme - a survey on the quality of care among patients with heart failure in Europe. Part 1: patient characteristics and diagnosis. Eur Heart J.

[14] Tracking Heart Disease and Stroke in Canada. 2009 . Available from: http://www.phac-aspc.gc.ca/publicat/2009/cvd-avc/pdf/cvd-avs-2009-eng.pdf

[15] National Clinical Guideline Centre (2010). Chronic heart failure: the management of chronic heart failure in adults in primary and secondary care.

[16] Butler J, Braunwald E, Gheorghiade M (2014). Recognizing worsening chronic heart failure as an entity and an end point in clinical trials. JAMA.

[17] Solomon SD, Dobson J, Pocock SJ, Skali H, McMurray JJV, Granger CB, Yusuf S, Swedberg KB, Young JB, Michelson EL, Pfeffer MA (2007). Candesartan in Heart failure Assessment of Reduction in Mortality and morbidity CHARM Investigators. Influence of nonfatal hospitalization for heart failure on subsequent mortality in patients with chronic heart failure. Circulation.

[18] Gheorghiade M, Vaduganathan M, Fonarow GC, Bonow RO (2013). Rehospitalization for heart failure: problems and perspectives. J Am Coll Cardiol.

[19] O’Connor CM, Abraham WT, Albert NM, Clare R, Gattis Stough W, Gheorghiade M, Greenberg BH, Young JB, Yancy CW, Fonarow GC (2008). Predictors of mortality after discharge in patients hospitalized with heart failure: an analysis from the Organized Program to Initiate Lifesaving Treatment in Hospitalized Patients with Heart Failure (OPTIMIZE-HF). Am Heart J.

[20] Skali H (2014). Prognosis and response to therapy of first inpatient and outpatient heart failure event in a heart failure clinical trial: MADIT-CRT. Eur J Heart Fail.

[21] Senni M, Gavazzi A, Oliva F (2014). In-hospital and 1-year outcomes of acute heart failure patients according to presentation (de novo vs. worsening) and ejection fraction. Results from IN-HF Outcome Registry. Int J Cardiol.

[22] Rosamond W, Flegal K, Friday G (2007). Heart disease and stroke statistics-2007 update: a report from the American Heart Association Statistics Committee and Stroke Statistics Subcommittee. Circulation.

[23] Eshel N, Raz R, Chodick G, Guindy M (2013). Characteristics of the elderly who do not visit primary care physicians. Isr J Health Policy Res.

[24] Stock SA, Stollenwerk B, Redaelli M, Civello D, Lauterbach KW (2008). Sex differences in treatment patterns of six chronic diseases: an analysis from the German statutory health insurance. J Womens Health (Larchmt).

[25] Chodick G (2003). The epidemiology of diabetes in a large Israeli HMO. Eur J Epidemiol.

[26] Shalev V (2011). The use of an automated patient registry to manage and monitor cardiovascular conditions and related outcomes in a large health organization. Int J Cardiol.

[27] Hoover DR, Crystal S, Kumar R, Sambamoorthi U, Cantor JC (2002). Medical Expenditures during the Last Year of Life: Findings from the 1992–1996 Medicare Current Beneficiary Survey. Health Serv Res.

[28] Felker GM (2011). Diuretic strategies in patients with acute decompensated heart failure. N Engl J Med.

[29] Heymann AD (2007). Description of a diabetes disease register extracted from a central database. Harefuah.

[30] Chodick G (2010). The direct medical cost of cardiovascular diseases, hypertension, diabetes, cancer, pregnancy and female infertility in a large HMO in Israel. Health Policy.

[31] Burck L, Tsibel N (2013). Characterization and Classification of Geographical Units by the Socio-Economic Level of the Population.

[32] Ministry of Health: Price List. http://www.health.gov.il/subjects/finance/taarifon/pages/pricelist.aspx

[33] Gertner Institute Advanced Technology Call Center. http://www.gertnerinst.org.il/e/health_society/chronic_morbidity_in_community/MOMA/

[34] Kalter-Leibovici O, Freimark D, Freedman L, Ziv A, Murad H, Benderly M, Friedman N, Kaufman G, Silver H (2014). Community Disease Management Program in Patients with Heart Failure: A Randomized Controlled Trial. Circulation.

[35] Peinado I, Villalba E, Mansoa F, Sánchez A. JRC Science and Policy Reports. Strategic Intelligence Monitor on Personal Health Systems Phase 3 (SIMPHS3): MOMA and Maccabi Healthcare Services (Israel) Case Study Report. 2015. Report EUR 27261 EN.

[36] Poppe KK, Squire IB, Whalley GA, Køber L, McAlister FA, McMurray JJ, Pocock S, Earle NJ, Berry C, Doughty RN (2013). Meta-Analysis Global Group in Chronic Heart Failure. Known and missing left ventricular ejection fraction and survival in patients with heart failure: a MAGGIC meta-analysis report. Eur J Heart Fail.

[37] Esse T, Serna O, Chitnis A, Johnson M, Fernandez N (2013). Quality compensation programs: are they worth all the hype? A comparison of outcomes within a Medicare advantage heart failure population. J Manag Care Pharm.

[38] Layton TJ, Ryan AM (2015). Higher Incentive Payments in Medicare Advantage’s Pay-for-Performance Program Did Not Improve Quality But Did Increase Plan Offerings. Health Serv Res.

[39] Ryan A, Sutton M, Doran T (2014). Does winning a pay-for-performance bonus improve subsequent quality performance? Evidence from the Hospital Quality Incentive Demonstration. Health Serv Res.

